# Exploiting Mitochondria by Triggering a Faulty Unfolded Protein Response Leads to Effective Cardioprotection

**DOI:** 10.7150/ijms.100523

**Published:** 2025-01-01

**Authors:** Yang Shen, Xin Gao, Ying Xiang, Hao Zhou, Hang Zhu, Qiang Wu, Jinfeng Liu

**Affiliations:** 1 Guang'anmen Hospital, China Academy of Chinese Medical Sciences, Beijing 100053, China.; 2 Department of Cardiology, School of Medicine, South China University of Technology, Guangzhou 510006, China.; 3 Senior Department of Cardiology, the Sixth Medical Center, Chinese PLA General Hospital, Beijing 100048, China.; 4 Outpatient Department of the Sixth Medical Center of the General Hospital of the People's Liberation Army, China.; 5 Senior Department of Traditional Chinese Medicine, the Sixth Medical Center of PLA General Hospital, China.

**Keywords:** FUNDC1, ATF5, mito-UPR, mitochondria.

## Abstract

This study investigates the role of Fundc1 in cardiac protection under high-altitude hypoxic conditions and elucidates its underlying molecular mechanisms. Using cardiomyocyte-specific *Fundc1* knockout (*Fundc1^CKO^*) mice, we demonstrated that *Fundc1* deficiency exacerbates cardiac dysfunction under simulated high-altitude hypoxia, manifesting as impaired systolic and diastolic function. Mechanistically, we identified that Fundc1 regulates cardiac function through the mitochondrial unfolded protein response (mito-UPR) pathway. *Fundc1* deficiency led to significant downregulation of multiple mito-UPR-related factors, including ATF5, Chop, and PITRM1. Further investigation revealed that Fundc1 deficiency results in increased cardiomyocyte apoptosis, calcium dysregulation, reduced cell viability, and impaired mitochondrial function, characterized by decreased ATP production, reduced membrane potential, and increased ROS production. Notably, activation of mito-UPR with oligomycin significantly ameliorated these cardiac abnormalities in Fundc1-deficient mice. We identified ATF5 as a key downstream effector of Fundc1, as ATF5 overexpression effectively reversed cardiac dysfunction and restored mito-UPR-related gene expression in Fundc1-deficient hearts. Additionally, we discovered that Fundc1-mediated cardioprotection involves regulation of mitophagy, where its activation improved cardiac function and mitochondrial homeostasis in Fundc1-deficient mice. Our findings reveal a novel Fundc1-ATF5-mito-UPR axis in cardioprotection against high-altitude hypoxia and highlight the crucial role of mitophagy in this protective mechanism, providing new insights into potential therapeutic strategies for high-altitude heart disease.

## Introduction

High-altitude hypoxia-induced myocardial injury is a significant clinical problem, causing cardiac dysfunction and increased mortality 1-3. The heart's high oxygen demand makes it vulnerable to hypoxia, triggering mitochondrial dysfunction, oxidative stress, calcium dysregulation, and apoptosis, ultimately impairing cardiac function 4-7. While traditional therapies focus on symptom management and oxygen, research now emphasizes mitochondrial quality control pathways as crucial for cardiomyocyte survival under hypoxic stress.

The mitochondrial unfolded protein response (mito-UPR) is crucial for maintaining mitochondrial proteostasis under stress, including hypoxia 8-11. This pathway, activated by mitochondrial protein misfolding, triggers transcriptional upregulation of chaperones, proteases, and other quality control factors via transcription factors like ATF5 12-16. The mito-UPR protects cardiomyocytes from stress, including ischemia-reperfusion and oxidative damage, by enhancing mitochondrial function, reducing oxidative stress, and maintaining calcium homeostasis 17-20. However, its regulation and role in cardiac protection during high-altitude hypoxia remain unclear.

Fundc1, a mitochondrial outer membrane protein, is a key regulator of mitochondrial quality control and adaptation to high-altitude hypoxia 21-25. Initially identified as a mitophagy receptor, Fundc1 maintains mitochondrial function through selective autophagic degradation of damaged mitochondria 26-29. Its activity, regulated by hypoxia and stress signals, is crucial for cardiomyocyte protection 30-34. While Fundc1's role extends beyond mitophagy, its complete regulatory function in cardiac protection during high-altitude hypoxia requires further investigation.

The interplay between the mito-UPR and Fundc1-mediated protection is crucial for understanding cardiac adaptation to high-altitude hypoxia. These quality control mechanisms appear interconnected: mito-UPR activation influences mitochondrial dynamics regulated by Fundc1, while Fundc1-mediated mitophagy may impact mito-UPR activation. This bidirectional relationship, where Fundc1 may regulate mito-UPR components like ATF5 and mito-UPR activation influences FUNDC1-mediated mitophagy, offers potential therapeutic targets for high-altitude-induced cardiac injury.

## Methods

### Ethical statement

This study was performed in strict accordance with the recommendations in the Guide for the Care and Use of Laboratory Animals at China Academy of Chinese Medical Sciences. The animal study was approved by the Institutional Animal Care and Use Committee of China Academy of Chinese Medical Sciences (No. 241124).

### Animal models

This study used cardiomyocyte-specific Fundc1 knockout (Fundc1^CKO^) and control Fundc1^flox^ mice 35, generated as previously described. Mice were housed under standard 12-hour light/dark cycles with free access to food and water. Following acclimatization, mice were exposed to simulated hypobaric hypoxia (5000m; 53.9 kPa, 11.2 kPa O₂, 24.9°C, 23.4% humidity) for 4 weeks in a chamber with a 10 m/s ascent/descent rate and 93.5 m³/h ventilation, maintaining a near 12:12 light:dark cycle. Food and water were provided ad libitum, and chamber maintenance occurred daily between 8:00 and 9:00 AM 36.

### Echocardiography

Cardiac function was assessed by echocardiography (high-resolution ultrasound) in isoflurane-anesthetized mice on a heated platform 37. Left ventricular ejection fraction (LVEF), fractional shortening (LVFS), diastolic function (E/A, E'/E), and structural parameters (LVESD, LVEDD) were measured from parasternal long- and short-axis views. Data, averaged over three cardiac cycles, were analyzed by a blinded operator using specialized software 38.

### Quantitative PCR (qPCR)

Cardiac tissue RNA was extracted using TRIzol, quality-checked with a NanoDrop, and reverse transcribed (1 µg input) using a high-capacity cDNA kit. qPCR (SYBR Green) was performed for mito-UPR-related genes with GAPDH as a control 38, 39. Cycling conditions were 95°C for 10 minutes followed by 40 cycles of 95°C for 15 seconds and 60°C for 1 minute. Relative expression was calculated using the 2^-ΔΔCt^ method 40.

### Western blots

Proteins were extracted from heart tissues or cellular samples using RIPA buffer with protease and phosphatase inhibitors, quantified by Bradford assay, and 30 µg per sample were separated on 10% SDS-PAGE and transferred to PVDF membranes 41. Membranes were blocked (5% milk/TBST), incubated overnight with primary antibodies, then with HRP-conjugated secondary antibodies. Detection used ECL, and bands were normalized to β-actin 42.

### Cell viability assay

Cardiomyocyte viability under simulated high-altitude hypoxia was assessed by MTT assay. Cells (1×10⁴/well) were treated, then incubated with MTT (10 µL, 5 mg/mL) for 4 hours 43. Formazan crystals were dissolved in DMSO (100 µL), and absorbance measured at 570 nm. Viability was expressed as a percentage of the control 44.

### Apoptosis assays

Apoptosis was assessed by caspase-3 activity (p-nitroaniline release measured at 405 nm) and TUNEL staining 45. Fixed and permeabilized cardiomyocytes were TUNEL-stained, nuclei counterstained with DAPI, and TUNEL-positive cells counted (≥500 cells/sample) via fluorescence microscopy 46.

### Calcium homeostasis

Intracellular calcium levels were measured using Fluo-4 AM (5 µM, 30 minutes, 37°C). After washing, fluorescence intensity was measured microscopically, and images analyzed to quantify calcium levels under hypoxia 47.

### JC-1 staining

Mitochondrial membrane potential was measured using JC-1 (5 µM, 20 minutes, 37°C) 48. Following washes, red/green fluorescence ratios (aggregated/monomeric JC-1) were calculated from microscopy images to assess mitochondrial health 49.

### Reactive Oxygen Species (ROS) levels

ROS levels were measured using DCFDA (10 µM, 30 minutes, 37°C). Post-wash, fluorescence was measured (Ex/Em: 485/535 nm) and expressed as relative fluorescence units, indicating oxidative stress 50.

### ATP production

ATP levels were quantified using a luminescence-based assay 51. Cardiomyocyte lysates were reacted with luciferase reagent, and luminescence, proportional to ATP concentration, was measured. ATP levels (nmol/mg protein) were calculated against a standard curve 52, 53.

### Mitochondrial enzyme activity

Cytochrome c oxidase (COX) activity in isolated cardiac mitochondria was measured spectrophotometrically by monitoring reduced cytochrome c oxidation at 550 nm and expressed as units/mg protein 54, 55.

### Statistical analysis

Data are presented as mean ± SD. Statistical analysis (GraphPad Prism) used Student's t-test or one-way ANOVA with Tukey's post-hoc test. Significance was set at p < 0.05.

## Results

### Fundc1 deficiency impacts cardiac function and the expression levels of the mito-UPR

**Figure 1:**
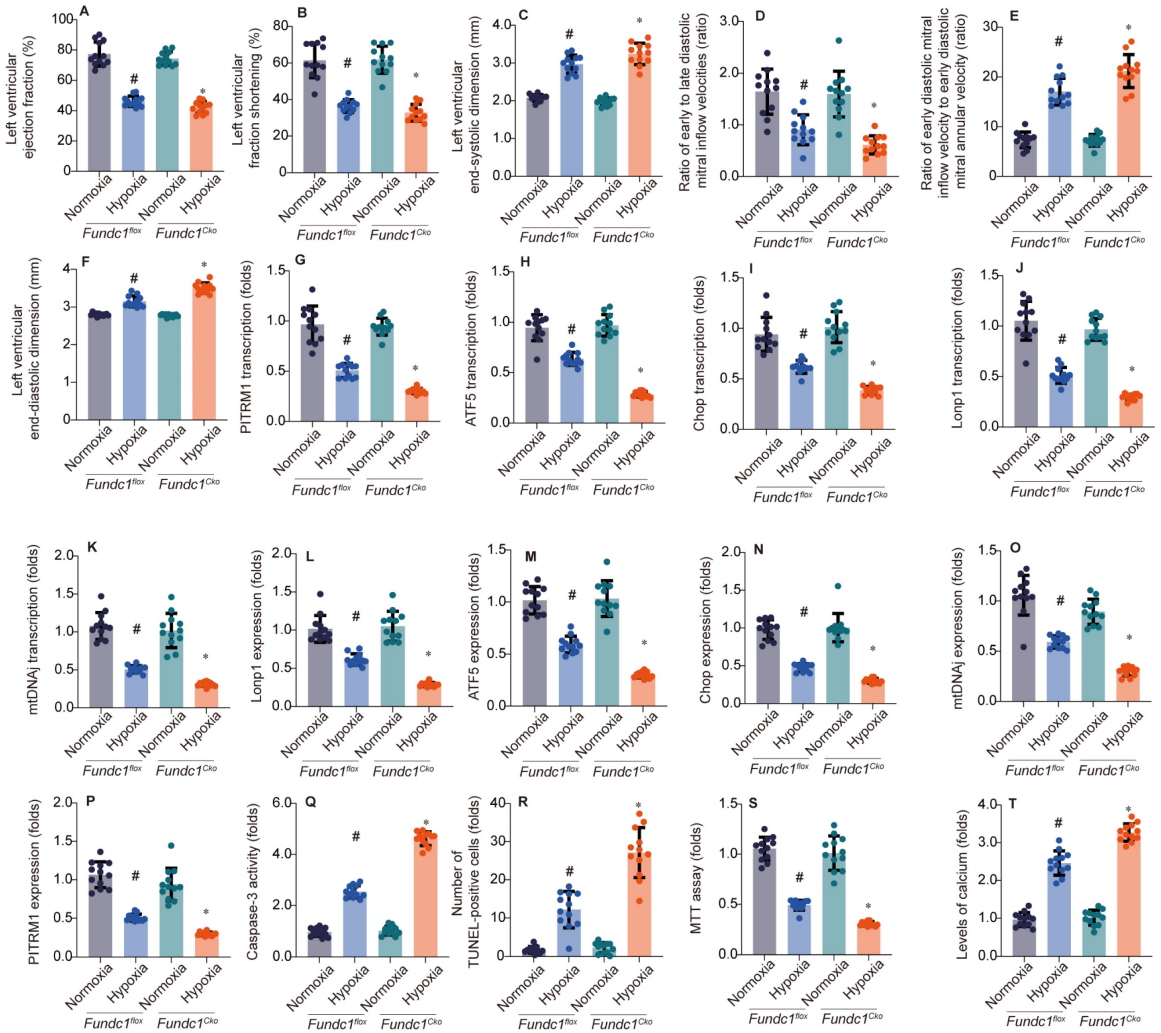
Fundc1 deficiency exacerbates hypoxia-induced cardiac dysfunction. Echocardiography showed FUNDC1 deficiency (Fundc1^CKO^) worsened high-altitude hypoxia-induced cardiac dysfunction compared to controls (Fundc1^flox^), affecting LVEF (A), LVFS (B), LVESD (C), E/A ratio (D), E/e' ratio (E), and LVEDD (F). (*p < 0.05 vs. normoxia; #p < 0.05 vs. Fundc1^flox^ under the same condition). Fundc1 deficiency alters the transcriptional response to hypoxia in the heart. Mitophagy and mitochondrial biogenesis marker mRNA expression (PITRM1 (G), ATF5 (H), Chop (I), Lonp1 (J), mtDNAJ (K)) were altered in FUNDC1-deficient (Fundc1^CKO^) mice compared to controls (Fundc1^flox^) under high-altitude hypoxia. (*p < 0.05 vs. normoxia; #p < 0.05 vs. Fundc1 under the same condition). Fundc1 deficiency alters the protein expression of mitophagy and mitochondrial biogenesis markers in response to hypoxia. Quantification (L-P) of Lonp1 (L), ATF5 (M), Chop (N), mtDNAJ (O), and PITRM1 (P) protein expression in Fundc1^flox^ and Fundc1^CKO^ mice under normoxic and high-altitude hypoxic conditions revealed alterations in FUNDC1-deficient mice. (*p < 0.05 vs. normoxia; #p < 0.05 vs. Fundc1^flox^). Fundc1 deficiency exacerbates hypoxia-induced cardiomyocyte apoptosis and dysfunction *in vitro*. FUNDC1 deficiency (Fundc1^CKO^) increased cardiomyocyte apoptosis (caspase-3 activity (Q), TUNEL (R)) and reduced viability (MTT assay (S)) under high-altitude hypoxia compared to controls (Fundc1^flox^). Intracellular calcium levels were also elevated in Fundc1^CKO^ cardiomyocytes (T). (*p < 0.05 vs. normoxia; #p < 0.05 vs. Fundc1^flox^).

To investigate Fundc1's role in hypoxia-induced myocardial injury, we compared cardiac function in *Fundc1^flox^* (control) and *Fundc1^CKO^* (knockout) mice under normoxia and simulated high-altitude hypoxia. *Fundc1^CKO^* mice exhibited greater systolic (LVEF, LVFS), diastolic (E/A, E'/E), and structural (LVESD, LVEDD) impairments (Fig. 1A-F), demonstrating Fundc1's protective role against high-altitude hypoxia-induced cardiac damage.

Investigating Fundc1's cardioprotective mechanism, we examined the mito-UPR. qPCR (Fig. 1G-K) and Western blot (Fig. 1L-P) analyses revealed that Fundc1 deficiency dysregulates mito-UPR-related factors at both transcriptional and protein levels under simulated high-altitude hypoxia. Specifically, Fundc1 deficiency reduced expression of transcription factors (ATF5, Chop), proteases (Lonp1), chaperones (mtHsp70/mtDNAj), and enzymes (PITRM1), indicating Fundc1's protective role via mito-UPR modulation.

To understand Fundc1 deficiency's impact on cardiomyocyte survival, we assessed apoptosis and calcium homeostasis under simulated high-altitude hypoxia (Fig. 1Q-T). Fundc1 deficiency increased caspase-3 activity and TUNEL-positive cells, reduced cell viability, and elevated intracellular calcium. These findings, correlating with mito-UPR dysregulation, underscore Fundc1's cardioprotective role via the Fundc1/mito-UPR axis. By maintaining mito-UPR function, Fundc1 mitigates hypoxia-induced apoptosis and calcium dysregulation, preserving cardiac function.

### Fundc1 protects heart function by activating mito-UPR

**Figure 2:**
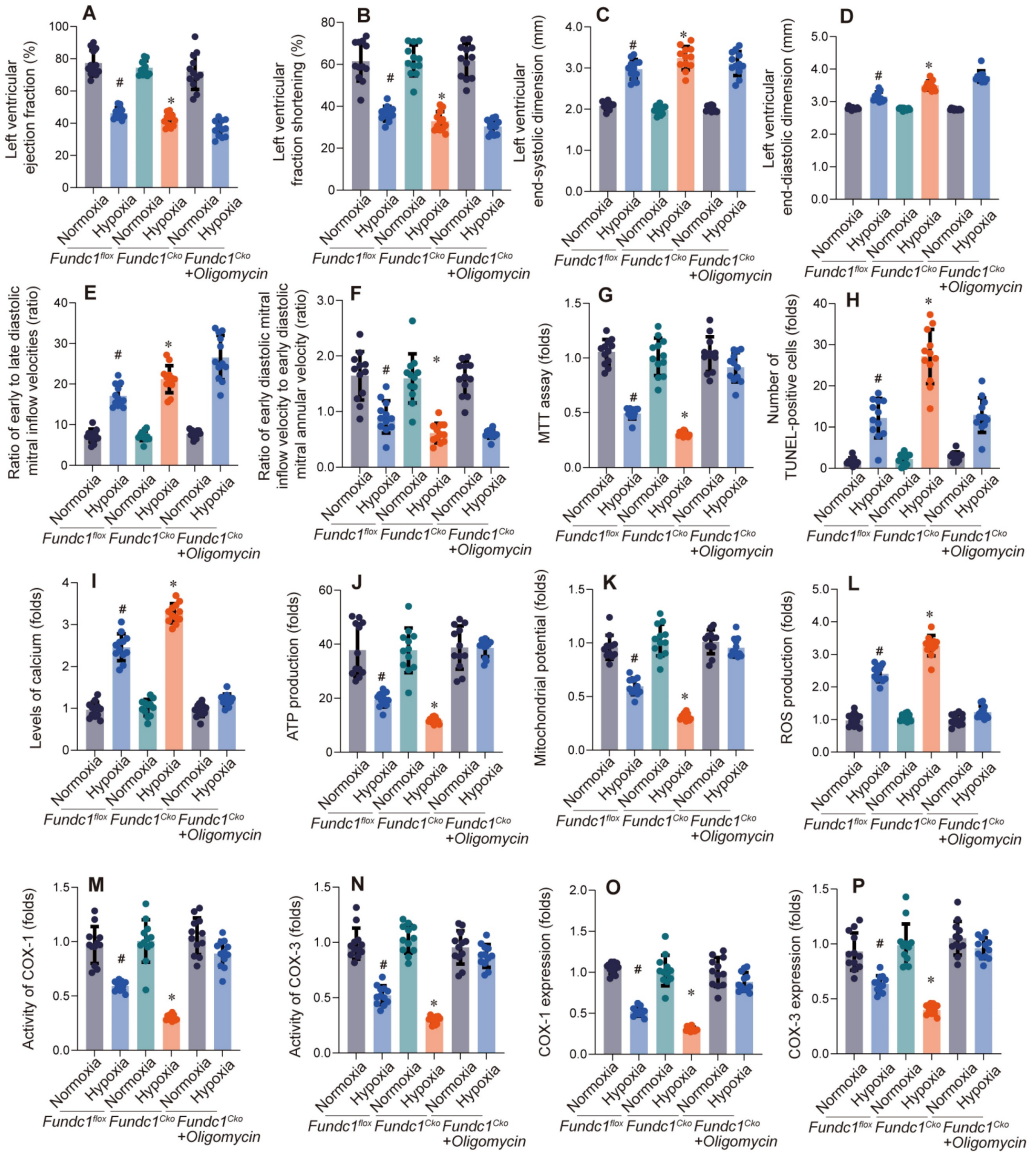
** Oligomycin treatment rescues hypoxia-induced cardiac dysfunction in Fundc1^CKO^ mice.** Echocardiography assessed cardiac function (LVEF (A), LVFS (B), LVESD (C), LVEDD (D), E/A ratio (E), E/e' ratio (F)) in Fundc1^flox^ and Fundc1^CKO^ mice under normoxic and high-altitude hypoxic conditions, with and without oligomycin. FUNDC1 deficiency worsened hypoxia-induced dysfunction. (*p < 0.05 vs. normoxia; #p < 0.05 vs. Fundc1^flox^). Oligomycin treatment rescues hypoxia-induced cardiomyocyte dysfunction and apoptosis *in vitro*. The effects of oligomycin treatment on cardiomyocyte viability (MTT assay (G)), apoptosis (TUNEL staining (H)), intracellular calcium (I), and ATP production (J) were assessed in Fundc1^flox^ and Fundc1^CKO^ cardiomyocytes under normoxic and high-altitude hypoxic conditions. FUNDC1 deficiency worsened hypoxia-induced changes in these parameters. (*p < 0.05 vs. normoxia; #p < 0.05 vs. Fundc1^flox^). Oligomycin treatment rescues hypoxia-induced mitochondrial dysfunction in Fundc1^CKO^ cardiomyocytes. Mitochondrial function was assessed in Fundc1^flox^ and Fundc1^CKO^ cardiomyocytes under normoxic and high-altitude hypoxic conditions with and without oligomycin. FUNDC1 deficiency impacted mitochondrial membrane potential (K), ROS production (L), COX activity (COX-1 (M), COX-3 (N)), and COX mRNA/protein expression (COX-1 (O), COX-3 (P)). (*p < 0.05 vs. normoxia; #p < 0.05 vs. Fundc1^flox^).

Having previously shown that Fundc1 deficiency downregulates mito-UPR factors *in vitro*, we activated the mito-UPR *in vivo* (Fig. 2A-F) to validate its role in Fundc1-mediated cardioprotection against Fundc1-deficiency-induced cardiac dysfunction.

Activating the mito-UPR with oligomycin (Figure 2) improved cardiac function in Fundc1-deficient mice under simulated high-altitude hypoxia, including improved systolic function (LVEF, LVFS), reduced ventricular dilation (LVEDD, LVESD), and improved diastolic function (E/A, E'/E).

These findings, consistent with FUNDC1's regulation of the mito-UPR, reinforce the importance of the Fundc1/mito-UPR axis in the cardiomyocyte response to high-altitude hypoxia. The observation that mito-UPR activation can compensate for Fundc1 deficiency's effects highlights the mito-UPR's role in Fundc1-mediated cardioprotection and offers a basis for new ischemic heart disease therapies.

Having established Fundc1's cardioprotective role via mito-UPR regulation, we investigated the effects of Fundc1 deficiency and mito-UPR activation on cardiomyocyte viability, apoptosis, and calcium homeostasis (Fig. 2G-J). Fundc1 deficiency increased susceptibility to reduced viability, apoptosis, calcium dyshomeostasis, and impaired energy metabolism under simulated high-altitude hypoxia. Mito-UPR activation with oligomycin mitigated these effects. These cellular findings explain the whole-heart functional changes and corroborate the molecular-level mito-UPR expression alterations, further emphasizing the Fundc1-mito-UPR axis's importance in cardiomyocyte protection.

To understand Fundc1 deficiency's impact on mitochondrial function, we conducted experiments presented in Figure 2K-P. Fundc1 deficiency caused mitochondrial dysfunction under simulated high-altitude hypoxia, including decreased membrane potential, increased ROS, reduced enzyme activity, and decreased protein expression. Mito-UPR activation with oligomycin improved these abnormalities. These mitochondrial findings explain the observed cellular defects and corroborate the molecular changes in mito-UPR factors, highlighting the Fundc1/mito-UPR axis's importance in protecting cardiac function during high-altitude hypoxia.

### Overexpression of ATF5 can improve cardiac function, restore mito-UPR activity, myocardial viability, mitochondrial structure and function

**Figure 3:**
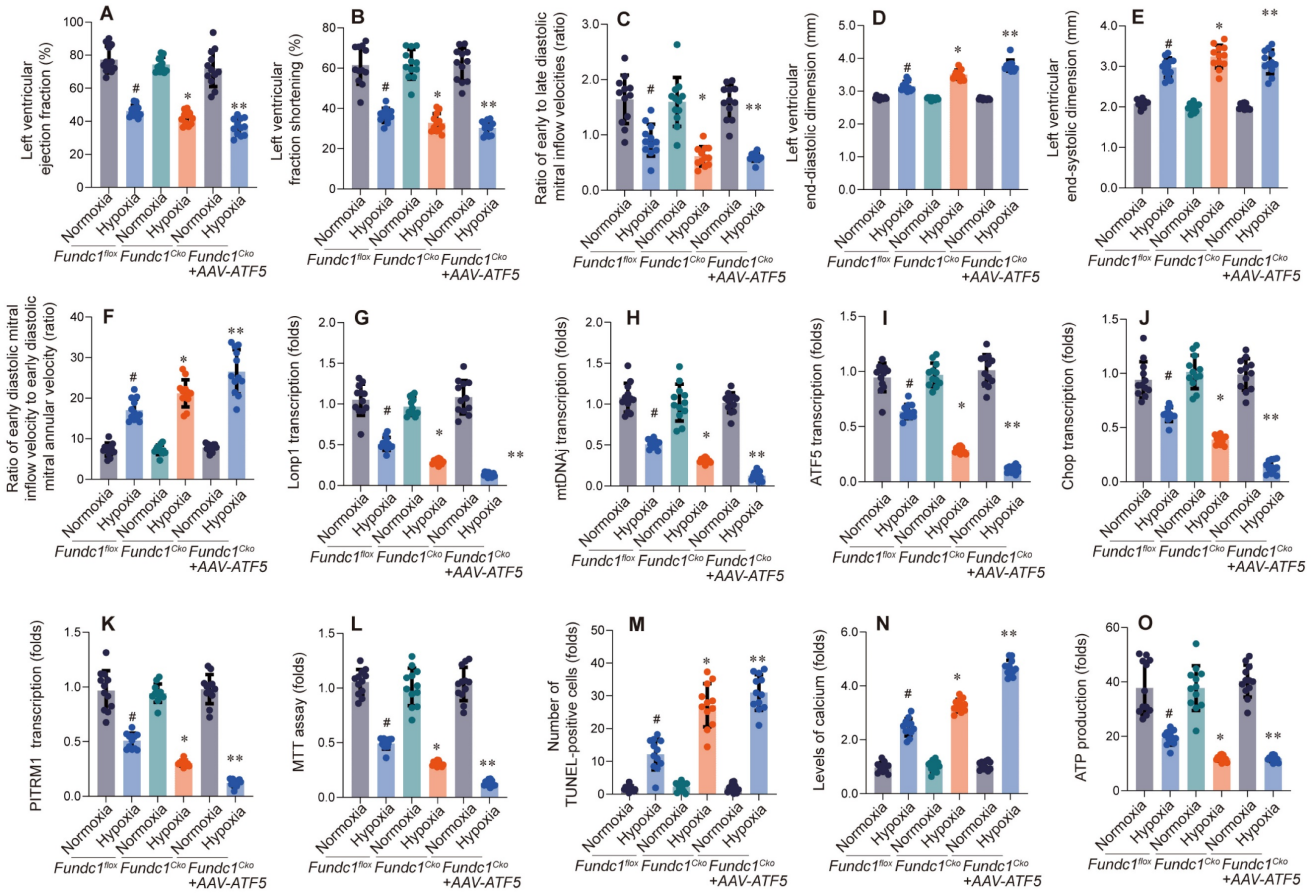
** ATF5 overexpression rescues hypoxia-induced cardiac dysfunction in Fundc1^CKO^ mice.** AAV-mediated ATF5 overexpression improved cardiac function in Fundc1^CKO^ mice under high-altitude hypoxia, as measured by LVEF (A), LVFS (B), E/A ratio (C), LVEDD (D), LVESD (E), and E/e' ratio (F). (*p < 0.05 vs. normoxia; #p < 0.05 vs. Fundc1^flox^; **p < 0.05 vs. Fundc1^CKO^ hypoxia). ATF5 overexpression rescues the altered transcriptional response to hypoxia in Fundc1^CKO^ hearts. AAV-ATF5 overexpression modulated mitophagy and mitochondrial biogenesis marker mRNA expression (Lonp1 (G), mtDNAJ (H), ATF5 (I), Chop (J), PITRM1 (K)) in Fundc1^CKO^ mice under high-altitude hypoxia. (*p < 0.05 vs. normoxia; #p < 0.05 vs. Fundc1^flox^; **p < 0.05 vs. Fundc1^CKO^ hypoxia). ATF5 overexpression rescues hypoxia-induced cardiomyocyte dysfunction and apoptosis *in vitro*. AAV-ATF5 overexpression improved cell viability (MTT assay (L)), reduced apoptosis (TUNEL staining (M)), decreased intracellular calcium levels (N), and increased ATP production (O) in Fundc1^CKO^ cardiomyocytes under high-altitude hypoxia. (*p < 0.05 vs. normoxia; #p < 0.05 vs. Fundc1^flox^; **p < 0.05 vs. Fundc1^CKO^ hypoxia).

Having shown that Fundc1 deficiency downregulates mito-UPR factors, especially ATF5, we hypothesized ATF5 is a key downstream effector of Fundc1. To test this, we overexpressed ATF5 (Figure 3) to determine if it could rescue FUNDC1-deficiency-induced cardiac dysfunction.

Overexpressing ATF5 (Fig. 3A-F) improved cardiac function in Fundc1-deficient mice, enhancing systolic function (LVEF, LVFS), diastolic function (E/A, e'/a'), and attenuating ventricular remodeling (LVEDD, LVESD). These findings, correlating with mito-UPR dysregulation, suggest ATF5 is a crucial downstream effector of FUNDC1, mediating cardioprotection against high-altitude hypoxia by activating the mito-UPR.

To explore ATF5's role in cardioprotection, we investigated its impact on mito-UPR gene expression in Fundc1-deficient mice (Fig. 3G-K). qPCR analysis revealed that ATF5 overexpression partially restored expression of mito-UPR-related genes, suggesting improved mitochondrial function. These findings support ATF5 as a key downstream effector of Fundc1, mediating cardioprotection against high-altitude hypoxia by activating mito-UPR-related genes.

To assess ATF5's cardioprotective effects, we examined its impact on Fundc1-deficient cardiomyocytes under simulated high-altitude hypoxia (Fig. 3L-O). ATF5 overexpression enhanced metabolic activity, reduced apoptosis, maintained calcium homeostasis, improved mitochondrial function, and increased ATP production.

### The impact of Fundc1-mediated mitophagy on myocardial injury under high-altitude hypoxic conditions

**Figure 4:**
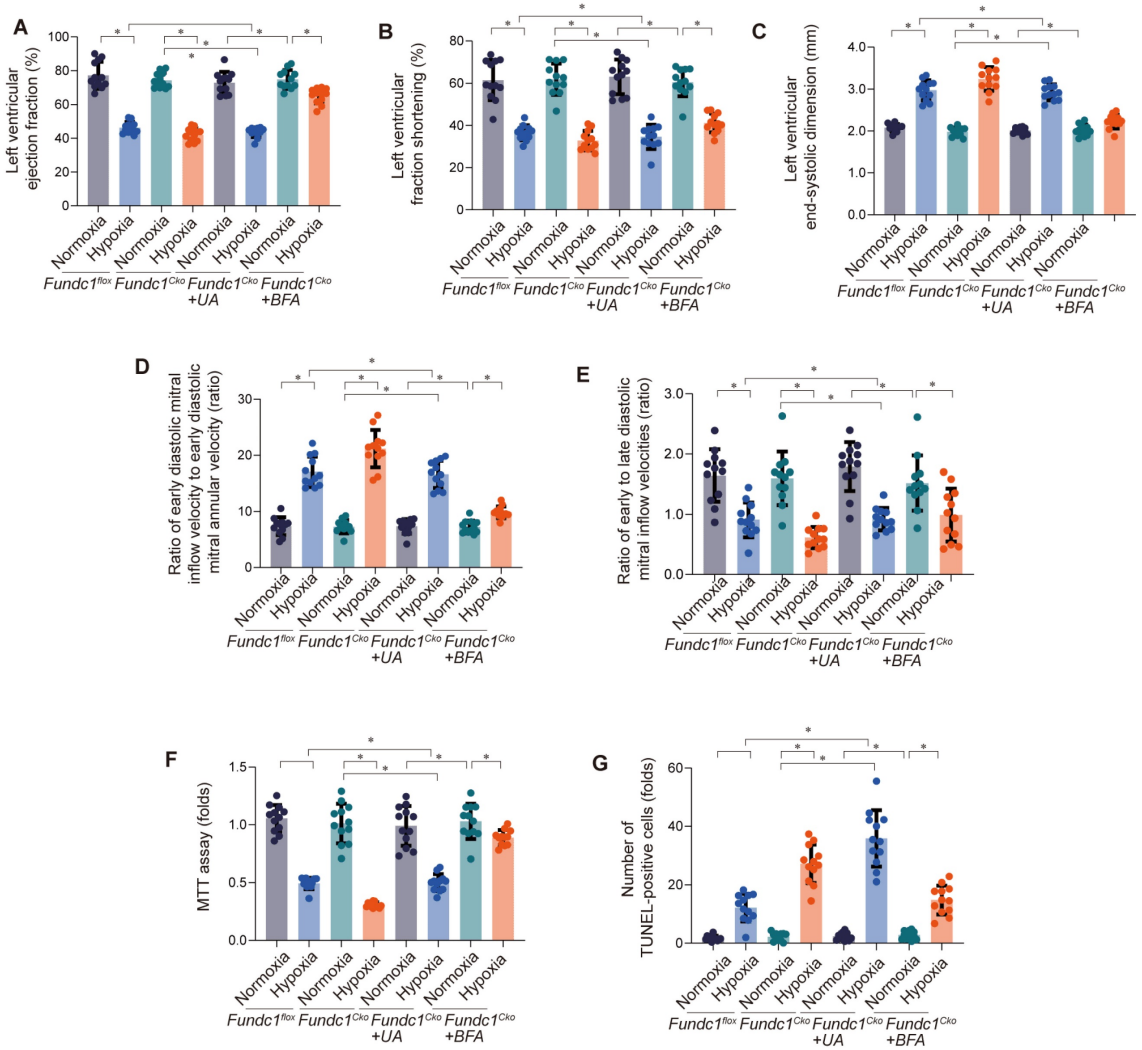
** Inhibition of mitophagy rescues hypoxia-induced cardiac dysfunction in Fundc1^CKO^ mice.** Mitophagy inhibition worsened cardiac function in Fundc1^flox^ and Fundc1^CKO^ mice under high-altitude hypoxia, as measured by LVEF (A), LVFS (B), LVESD (C), E/e' ratio (D), and E/A ratio (E). (*p < 0.05 vs. normoxia; #p < 0.05 vs. Fundc1^flox^). Inhibition of mitophagy rescues hypoxia-induced cardiomyocyte dysfunction and apoptosis *in vitro*. Mitophagy inhibition reduced cell viability (MTT assay (F)) and increased apoptosis (TUNEL staining (G)) in Fundc1^flox^ and Fundc1^CKO^ cardiomyocytes under high-altitude hypoxia. (*p < 0.05 vs. normoxia; #p < 0.05 vs. Fundc1^flox^).

Investigating Fundc1's cardioprotective mechanism via mito-UPR activation, we examined mitophagy's role (Fig. 4A-E). Fundc1 deficiency impaired systolic and diastolic function under simulated high-altitude hypoxia. Activating mitophagy improved cardiac function in Fundc1-deficient mice, while inhibiting it worsened dysfunction, confirming mitophagy's importance in Fundc1-mediated cardioprotection. Fundc1's benefits extended to preventing ventricular remodeling and preserving myocardial strain. These findings suggest Fundc1 promotes mitophagy to maintain mitochondrial quality and function, protecting the heart during high-altitude hypoxia.

Having observed Fundc1-mediated mitophagy's impact on cardiac function (Fig. 4A-E), we investigated its effect on cardiomyocyte viability and apoptosis (Fig. 4F-G). Fundc1 deficiency reduced viability and increased apoptosis under simulated high-altitude hypoxia. Activating mitophagy rescued these effects in Fundc1-deficient cells, while inhibiting mitophagy worsened them, confirming its role in cardiomyocyte protection. These findings demonstrate Fundc1's direct protective effect on cardiomyocyte survival, extending beyond global cardiac function.

These findings demonstrate Fundc1's critical role in preserving mitochondrial respiratory function through both mitophagy and metabolic modulation, protecting against energy depletion and oxidative stress. This supports developing cardioprotective strategies targeting Fundc1 and mitophagy.

## Discussion

This study reveals Fundc1's cardioprotective role during simulated high-altitude hypoxia. Fundc1-deficient mice exhibited exacerbated hypoxia-induced cardiac dysfunction, including impaired systolic and diastolic function and structural alterations, compared to controls 56. This highlights Fundc1's importance in maintaining cardiac performance via mitochondrial quality control 57. Furthermore, Fundc1 deficiency dysregulated mito-UPR factors, indicating Fundc1's role in modulating this protective response. Activating the mito-UPR partially rescued the dysfunction, suggesting the Fundc1/mito-UPR axis as a promising therapeutic target for high-altitude hypoxia-induced cardiac damage 58.

Furthermore, this study demonstrates Fundc1 deficiency leads to mitochondrial dysfunction, characterized by decreased ATP, increased ROS, and impaired respiratory chain activity. These findings highlight Fundc1-mediated mitophagy's role in mitochondrial quality control and cardiomyocyte survival 59, suggesting therapeutic potential for ischemic heart disease.

While this study advances understanding of Fundc1's cardioprotective role, limitations remain. The precise mechanisms of Fundc1's modulation of mito-UPR and mitophagy, and the broader implications of manipulating Fundc1's downstream effectors beyond ATF5, require further investigation 60, 61. Future studies should explore these pathways and their potential synergy with other cardioprotective mechanisms.

In conclusion, this research highlights Fundc1's critical role in protecting the heart during high-altitude hypoxia, suggesting the Fundc1/mito-UPR axis as a promising therapeutic target for managing cardiac dysfunction.

## Funding

This project is supported by the High Level Chinese Medical Hospital Promotion Project, Grant No: HLCMHPP2023111.

## References

[1] He S, Zhang Q, Wu F, Chen J, He S, Ji Z (2023). Influence of cigarettes on myocardial injury in healthy population after exposure to high altitude over 5000 m. Sci Total Environ.

[2] Mallet RT, Burtscher J, Richalet JP, Millet GP, Burtscher M (2021). Impact of High Altitude on Cardiovascular Health: Current Perspectives. Vasc Health Risk Manag.

[3] Zhao Y, Xiong W, Li C, Zhao R, Lu H, Song S (2023). Hypoxia-induced signaling in the cardiovascular system: pathogenesis and therapeutic targets. Signal Transduct Target Ther.

[4] Gallo G, Rubattu S, Volpe M (2024). Mitochondrial Dysfunction in Heart Failure: From Pathophysiological Mechanisms to Therapeutic Opportunities. Int J Mol Sci.

[5] Zhou B, Tian R (2018). Mitochondrial dysfunction in pathophysiology of heart failure. J Clin Invest.

[6] Peoples JN, Saraf A, Ghazal N, Pham TT, Kwong JQ (2019). Mitochondrial dysfunction and oxidative stress in heart disease. Exp Mol Med.

[7] Mialet-Perez J, Belaidi E (2024). Interplay between hypoxia inducible Factor-1 and mitochondria in cardiac diseases. Free Radic Biol Med.

[8] Inigo JR, Chandra D (2022). The mitochondrial unfolded protein response (UPR(mt)): shielding against toxicity to mitochondria in cancer. J Hematol Oncol.

[9] Sutandy FXR, Gößner I, Tascher G, Münch C (2023). A cytosolic surveillance mechanism activates the mitochondrial UPR. Nature.

[10] Zhu L, Luo X, Fu N, Chen L (2021). Mitochondrial unfolded protein response: A novel pathway in metabolism and immunity. Pharmacol Res.

[11] Naresh NU, Haynes CM (2019). Signaling and Regulation of the Mitochondrial Unfolded Protein Response. Cold Spring Harb Perspect Biol.

[12] Slavin MB, Kumari R, Hood DA (2022). ATF5 is a regulator of exercise-induced mitochondrial quality control in skeletal muscle. Mol Metab.

[13] Zhang X, Fan Y, Tan K (2024). A bird's eye view of mitochondrial unfolded protein response in cancer: mechanisms, progression and further applications. Cell Death Dis.

[14] Torres AK, Fleischhart V, Inestrosa NC (2024). Mitochondrial unfolded protein response (UPR(mt)): what we know thus far. Front Cell Dev Biol.

[15] Liu J, He X, Zheng S, Zhu A, Wang J (2022). The Mitochondrial Unfolded Protein Response: A Novel Protective Pathway Targeting Cardiomyocytes. Oxid Med Cell Longev.

[16] Wen L, Cao Y, Cheng Q, Li X, Pan L, Li L (2020). Objectively measured near work, outdoor exposure and myopia in children. Br J Ophthalmol.

[17] Kuznetsov AV, Javadov S, Margreiter R, Grimm M, Hagenbuchner J, Ausserlechner MJ (2019). The Role of Mitochondria in the Mechanisms of Cardiac Ischemia-Reperfusion Injury. Antioxidants (Basel).

[18] Zong Y, Li H, Liao P, Chen L, Pan Y, Zheng Y (2024). Mitochondrial dysfunction: mechanisms and advances in therapy. Signal Transduct Target Ther.

[19] Deng J, Wang D, Shi Y, Lin L, Gao W, Sun Y (2024). Mitochondrial unfolded protein response mechanism and its cardiovascular protective effects. Biomed Pharmacother.

[20] Zhang G, Wang X, Li C, Li Q, An YA, Luo X (2021). Integrated Stress Response Couples Mitochondrial Protein Translation With Oxidative Stress Control. Circulation.

[21] Bai X, Zhang Z, Li X, Yang Y, Ding S (2023). FUNDC1: An Emerging Mitochondrial and MAMs Protein for Mitochondrial Quality Control in Heart Diseases. Int J Mol Sci.

[22] Wu W, Lin C, Wu K, Jiang L, Wang X, Li W (2016). FUNDC1 regulates mitochondrial dynamics at the ER-mitochondrial contact site under hypoxic conditions. Embo j.

[23] Brischigliaro M, Sierra-Magro A, Ahn A, Barrientos A (2024). Mitochondrial ribosome biogenesis and redox sensing. FEBS Open Bio.

[24] Liu L, Feng D, Chen G, Chen M, Zheng Q, Song P (2012). Mitochondrial outer-membrane protein FUNDC1 mediates hypoxia-induced mitophagy in mammalian cells. Nat Cell Biol.

[25] Li Y, Liu Z, Zhang Y, Zhao Q, Wang X, Lu P (2018). PEDF protects cardiomyocytes by promoting FUNDC1-mediated mitophagy via PEDF-R under hypoxic condition. Int J Mol Med.

[26] Zhang W, Siraj S, Zhang R, Chen Q (2017). Mitophagy receptor FUNDC1 regulates mitochondrial homeostasis and protects the heart from I/R injury. Autophagy.

[27] Li G, Li J, Shao R, Zhao J, Chen M (2021). FUNDC1: A Promising Mitophagy Regulator at the Mitochondria-Associated Membrane for Cardiovascular Diseases. Front Cell Dev Biol.

[28] Uoselis L, Nguyen TN, Lazarou M (2023). Mitochondrial degradation: Mitophagy and beyond. Mol Cell.

[29] Li K, Xia X, Tong Y (2024). Multiple roles of mitochondrial autophagy receptor FUNDC1 in mitochondrial events and kidney disease. Front Cell Dev Biol.

[30] Zhang W (2021). The mitophagy receptor FUN14 domain-containing 1 (FUNDC1): A promising biomarker and potential therapeutic target of human diseases. Genes Dis.

[31] Wang S, Long H, Hou L, Feng B, Ma Z, Wu Y (2023). The mitophagy pathway and its implications in human diseases. Signal Transduct Target Ther.

[32] Huang K, Yang W, Shi M, Wang S, Li Y, Xu Z (2024). The Role of TPM3 in Protecting Cardiomyocyte from Hypoxia-Induced Injury via Cytoskeleton Stabilization. Int J Mol Sci.

[33] Kacimi R, Chentoufi J, Honbo N, Long CS, Karliner JS (2000). Hypoxia differentially regulates stress proteins in cultured cardiomyocytes: role of the p38 stress-activated kinase signaling cascade, and relation to cytoprotection. Cardiovasc Res.

[34] Yu W, Qin X, Zhang Y, Qiu P, Wang L, Zha W (2020). Curcumin suppresses doxorubicin-induced cardiomyocyte pyroptosis via a PI3K/Akt/mTOR-dependent manner. Cardiovasc Diagn Ther.

[35] Chen L, Tian Q, Shi Z, Qiu Y, Lu Q, Liu C (2021). Melatonin Alleviates Cardiac Function in Sepsis-Caused Myocarditis via Maintenance of Mitochondrial Function. Front Nutr.

[36] Wang X, Chen JD (2023). Therapeutic potential and mechanisms of sacral nerve stimulation for gastrointestinal diseases. J Transl Int Med.

[37] Liu Y, Liu Y, Ye S, Feng H, Ma L (2023). A new ferroptosis-related signature model including messenger RNAs and long non-coding RNAs predicts the prognosis of gastric cancer patients. J Transl Int Med.

[38] Chen L, Zhan CZ, Wang T, You H, Yao R (2020). Curcumin Inhibits the Proliferation, Migration, Invasion, and Apoptosis of Diffuse Large B-Cell Lymphoma Cell Line by Regulating MiR-21/VHL Axis. Yonsei Med J.

[39] Shao Y, Zhao T, Zhang W, He J, Lu F, Cai Y (2020). Presence of the apolipoprotein E-ε4 allele is associated with an increased risk of sepsis progression. Sci Rep.

[40] Li Y, Yao Z, Li Y, Yang Z, Li M, Chen Z (2023). Prognostic value of serum ammonia in critical patients with non-hepatic disease: A prospective, observational, multicenter study. J Transl Int Med.

[41] Cuny H, Bozon K, Kirk RB, Sheng DZ, Bröer S, Dunwoodie SL (2023). Maternal heterozygosity of Slc6a19 causes metabolic perturbation and congenital NAD deficiency disorder in mice. Dis Model Mech.

[42] Du Y, Li J, Dai Z, Chen Y, Zhao Y, Liu X (2024). Pyruvate kinase M2 sustains cardiac mitochondrial quality surveillance in septic cardiomyopathy by regulating prohibitin 2 abundance via S91 phosphorylation. Cell Mol Life Sci.

[43] Ding L, Lu S, Zhou Y, Lyu D, Ouyang C, Ma Z (2020). The 3' Untranslated Region Protects the Heart from Angiotensin II-Induced Cardiac Dysfunction via AGGF1 Expression. Mol Ther.

[44] Chang X, Lochner A, Wang HH, Wang S, Zhu H, Ren J (2021). Coronary microvascular injury in myocardial infarction: perception and knowledge for mitochondrial quality control. Theranostics.

[45] Peng Y, Wang Y, Zhou C, Mei W, Zeng C (2022). PI3K/Akt/mTOR Pathway and Its Role in Cancer Therapeutics: Are We Making Headway?. Front Oncol.

[46] Dou L, Lu E, Tian D, Li F, Deng L, Zhang Y (2023). Adrenomedullin induces cisplatin chemoresistance in ovarian cancer through reprogramming of glucose metabolism. J Transl Int Med.

[47] Deng Y, Wang H, Guo X, Jiang S, Cai J (2023). Long-term blood pressure outcomes of laparoscopic adrenalectomy in trHTN patients. J Transl Int Med.

[48] Lu Y, Lin Z, Wen L, Gao W, Pan L, Li X (2020). The Adaptation and Acceptance of Defocus Incorporated Multiple Segment Lens for Chinese Children. Am J Ophthalmol.

[49] Elefantova K, Lakatos B, Kubickova J, Sulova Z, Breier A (2018). Detection of the Mitochondrial Membrane Potential by the Cationic Dye JC-1 in L1210 Cells with Massive Overexpression of the Plasma Membrane ABCB1 Drug Transporter. Int J Mol Sci.

[50] Deng M, Wang M, Zhang Q, Jiang B, Yan L, Bian Y (2023). Point-of-care ultrasound-guided submucosal paclitaxel injection in tracheal stenosis model. J Transl Int Med.

[51] Jiang L, Chen T, Xiong L, Xu JH, Gong AY, Dai B (2020). Knockdown of m6A methyltransferase METTL3 in gastric cancer cells results in suppression of cell proliferation. Oncol Lett.

[52] Delgado-Camprubi M, Esteras N, Soutar MP, Plun-Favreau H, Abramov AY (2017). Deficiency of Parkinson's disease-related gene Fbxo7 is associated with impaired mitochondrial metabolism by PARP activation. Cell Death Differ.

[53] Chang X, Li Y, Cai C, Wu F, He J, Zhang Y (2022). Mitochondrial quality control mechanisms as molecular targets in diabetic heart. Metabolism.

[54] Kogot-Levin A, Saada A, Leibowitz G, Soiferman D, Douiev L, Raz I (2016). Upregulation of Mitochondrial Content in Cytochrome c Oxidase Deficient Fibroblasts. PLoS One.

[55] Chen X, Wang M, Yu K, Xu S, Qiu P, Lyu Z (2023). Chronic stress-induced immune dysregulation in breast cancer: Implications of psychosocial factors. J Transl Int Med.

[56] Huang Z, Yu P, Tang J (2020). Characterization of Triple-Negative Breast Cancer MDA-MB-231 Cell Spheroid Model. Onco Targets Ther.

[57] Suliman HB, Piantadosi CA (2016). Mitochondrial Quality Control as a Therapeutic Target. Pharmacol Rev.

[58] Gao WL, Li XH, Dun XP, Jing XK, Yang K, Li YK (2020). Grape Seed Proanthocyanidin Extract Ameliorates Streptozotocin-induced Cognitive and Synaptic Plasticity Deficits by Inhibiting Oxidative Stress and Preserving AKT and ERK Activities. Curr Med Sci.

[59] Atici AE, Crother TR, Noval Rivas M (2023). Mitochondrial quality control in health and cardiovascular diseases. Front Cell Dev Biol.

[60] Wang G, Fan Y, Cao P, Tan K (2022). Insight into the mitochondrial unfolded protein response and cancer: opportunities and challenges. Cell Biosci.

[61] Ji H, Wang J, Muid D, Song W, Jiang Y, Zhou H (2022). FUNDC1 activates the mitochondrial unfolded protein response to preserve mitochondrial quality control in cardiac ischemia/reperfusion injury. Cell Signal.

